# Supplementary material for: Transferring structural knowledge across cognitive maps in humans and models
Source: Nat Commun. 2020 Sep 22;11:4783. doi: 10.1038/s41467-020-18254-6 (PMC7508979; doi:10.1038/s41467-020-18254-6)
Supplement: Supplementary file 1 — Supplementary Information [file 41467_2020_18254_MOESM1_ESM.pdf]

Transferring structural knowledge across cognitive maps in humans and models

By Mark et al.

Supplementary information

Supplementary Methods: Estimating $p(\vec{O}|A_{sf}^{\theta}, \hat{B})$ and *B* using a variant of the Baum-Welch algorithm:

Learning from random walk: For each considered graph dimension, that belongs to one of the structural forms, we have estimated the transition matrix (A_{sf}^{θ}) using the rescaled basis set of that form. We then estimated, for each approximated transition matrix, the emission matrix *B* and the probability of the observations given these matrices, $p(\vec{O}|A_{sf}^{\theta}, \hat{B})$, using the Baum-Welch algorithm. The Baum-Welch algorithm estimates both the emission matrix and the transition matrix, here, we assumed that the transition matrix is known and therefore did not estimate it. We adapted the matlab routine 'hmmtrain' such that the transition matrix is given and we estimated *B* and $p(\vec{O}|A_{sf}^{\theta}, \hat{B})$.

Simulations details: During our simulations, the agents saw 150 pairs of pictures within each block. When the correct underlying structure was of a community structure, we averaged our results over 20 simulations, while when it was hexagonal grid we averaged over 15 simulations.

Initialization of parameters:

Hexagonal grid:

$$B_{z=1}(o=1)=1,\ B_{z=1}(o\neq 1)=0, B_{z\neq 1}(o=1)=0, B_{z=i}(o=j)=p_0\ i,j\neq 1 \quad (1)$$

$$p_0=\frac{1}{N_p-1} \quad (2)$$

Where *z* is a state and *o* is an observation, N_p is the number of observations.

Community structure:

Because of the symmetry of the community graph structure, the first picture (observation) can be a connecting node (therefore will assign arbitrarily to state 1) or a 'not connecting node' and therefore assign arbitrary to state 2. As there are 2 connecting nodes in a community the initial condition to the emission matrix is:

$$B_{z=1}(o=1)=\frac{2}{N_c}, B_{z=2}(o=1)=\frac{N_c-2}{N_c} \quad (3)$$

$$B_{z=1}(o\neq 1)=p_0\frac{N_c-2}{N_c}, B_{z\neq 1}(o=1)=\frac{2p_0}{N_c}, B_{z=i}(o=j)=p_0\ i,j\neq 1 \quad (4)$$

Where N_c is the community size.

The simulations were initiated in state 1.

Learning from pairs (Expectation – Maximization, EM): Learning from pairs that are randomly sampled from the graph, instead of a random walk (in which each pair has a picture from the previous pair), results in a process which is HMM of length 2. Therefore,

using the Baum-Welch is not relevant anymore. Instead, we estimated the emission matrix (*B*) and the likelihood, $p(\vec{O}|A^{\theta}_{sf},\hat{B})$, by the following EM algorithm:

Given a structural form and a graph dimension the transition matrix is approximated as before (A^{θ}_{sf}). For each particular transition matrix, we calculated the following EM steps until convergence:

(1) <u>Expectation step</u>:
In the following, $\vec{z}$ denotes the hidden states. B^{s-1} is the estimation of the emission matrix from the previous EM step (where *s* is the step).
The observation of each pair of pictures is independent from the previous pair, therefore the probability of the observations and hidden states is given by:

$$p(\vec{O},\vec{z}|A^{\theta}_{sf},B) = \prod_n p(O^n_1,O^n_2,z^n_1,z^n_2|A^{\theta}_{sf},B) \tag{5}$$

Where o^n_1 (o^n_2) is the first (second) observation in the *n's* pair, and z^n_1 (z^n_2) are the states of the first and second observation respectively.

Lets define:

$$\begin{aligned}\alpha_n(z^n_1=i,z^n_2=j) &\equiv p(O^n_1,O^n_2,z^n_1=i,z^n_2=j|A^{\theta}_{sf},B)\\ &= p(z^n_1=i)\cdot p(O^n_1|z^n_1=i,B)\cdot p(z^n_2=j|z^n_1=i,A^{\theta}_{sf})\\ &\cdot p(O^n_2|z^n_2=j,B) = \frac{1}{N}B_{z^n_1}(o^n_1)A_{ij}B_{z^n_2}(o^n_2)\end{aligned} \tag{6}$$

N is the number of nodes on the graph. Each pair is sampled randomly and uniformly therefore: $p(z^n_1)=\frac{1}{N}$. $B_{z^n_k}(o^n_k)=p(O^n_k|z^n_k)$ and

$$A_{ij} = p(z^n_2=i|z^n_1=j) = \begin{cases}\frac{1}{6} & if\ i\&j\ are\ neighbours\\ 0 & otherwise\end{cases} \tag{7}$$

and:

$$\log(p(\vec{O},\vec{z}|A^{\theta}_{sf},B) = \sum_n \log[p(O^n_1,O^n_2,z^n_1,z^n_2|A^{\theta}_{sf},B)] = \sum_n \log[\alpha_n(z^n_1,z^n_2)] = \sum_n\left(-logN + logB_{z^n_1}(o^n_1) + logA_{ij} + logB_{z^n_2}(o^n_2)\right) \tag{8}$$

The expected log likelihood of the data $(\vec{O},\vec{z})$ given the current model parameters (A^{θ}_{sf}), the previous estimation of the parameters (B^{s-1}) and the the distribution of the states $(\vec{z})$ conditioned on observation $(\vec{O})$ becomes:

$$Q(B,B^{s-1}) = \sum_{z\in Z} p(\vec{z}|\vec{O},A^{\theta}_{sf},B^{s-1})\log(p(\vec{O},\vec{z}|A^{\theta}_{sf},B) = \sum_{z\in Z} p(\vec{z}|\vec{O},A^{\theta}_{sf},B^{s-1})\sum_n \log[\alpha_n(z^n_1,z^n_2)] \tag{9}$$

And the likelihood of the current estimation of B is:

$$L(B^{s-1}) = p(\vec{O}|A_{sf}^{\theta}, B^{s-1}) = \prod_{n} p(O_1^n, O_2^n|A_{sf}^{\theta}, B^{s-1})$$

$$= \prod_{n} \sum_{i,j} \alpha_n^{s-1}(z_1^n = i, z_2^n = j) \tag{10}$$

Where $\alpha_n^{s-1}(z_1^n = i, z_2^n = j) = p\left(O_1^n, O_2^n, z_1^n = i, z_2^n = j|A_{sf}^{\theta}, B^{s-1}\right)$

On each expectation step we calculated: $\alpha_n^{s-1}(z_1^n = i, z_2^n = j)$ and $L(B^{s-1})$.

(2) <u>maximization step</u>:

We define a Lagrangian ,using Lagrange multiplier to satisfy the condition that $\sum_k B_i(k)$=1:

$$\hat{L}_{\lambda} = Q(B, B^{s-1}) - \sum_i \lambda_i (\sum_k B_i(k) - 1) \tag{11}$$

As we are maximizing in respect to B, we can keep only the following terms:

$$\tilde{L}_{\lambda} = \sum_{z \in Z} p\left(\vec{z}|\vec{O}, A_{sf}^{\theta}, B^{s-1}\right) \sum_n \left(logB_{z_1^n}(o_1^n) + logB_{z_2^n}(o_2^n)\right) - \sum_i \lambda_i (\sum_k B_i(k) - 1) = \sum_{i,j} \sum_n p\left(z_1^n = i|\vec{O}, A_{sf}^{\theta}, B^{s-1}\right) logB_i(O_1^n) + p(z_2^n = j|\vec{O}, A_{sf}^{\theta}, B^{s-1}) logB_j(O_2^n) - \sum_i \lambda_i (\sum_k B_i(k) - 1) \tag{12}$$

Maximizing in respect to $B_i\left(O_{1,2}^n = k\right)$ gives:

$$\frac{\partial \hat{L}_{\lambda}}{\partial B_i(k)} = \frac{\sum_n \left(p\left(z_1^n = i|\vec{O}, A_{sf}^{\theta}, B^{s-1}\right)\delta(o_1^n = k) + p\left(z_2^n = i|\vec{O}, A_{sf}^{\theta}, B^{s-1}\right)\right)\delta(o_2^n = k)\right)}{B_i(k)} - \lambda_k \tag{13}$$

For solving for $B_i(k)$ we define:

$$\tilde{\gamma}_n(i,j) \equiv p\left(z_1^n = i, z_2^n = j\middle|\vec{O}, A_{sf}^{\theta}, B^{s-1}\right) = \frac{p(z_1^n(i), z_2^n(j), \vec{O}|A_{sf}^{\theta}, B^{s-1})}{p(\vec{O}|A_{sf}^{\theta}, B^{s-1})} = \frac{p(z_1^n = i, z_2^n = j, O_1^n, O_2^n|A_{sf}^{\theta}, B^{s-1})\, p(O_{\sim n}|A_{sf}^{\theta}, B^{s-1})}{p(\vec{O}|A_{sf}^{\theta}, B^{s-1})} = \frac{\alpha_n^{s-1}(z_1^n = i, z_2^n = j)\, p(O_{\sim n}|A_{sf}^{\theta}, B^{s-1})}{p(\vec{O}|A_{sf}^{\theta}, B^{s-1})} \tag{14}$$

Where:

$$p(O_{\sim n}|A_{sf}^{\theta}, \hat{B}) = \prod_{m \sim n} \sum_{i,j} \alpha_m(z_1^m = i, z_2^m = j) \tag{15}$$

Therefore:

$$\gamma_n(i) \equiv p\left(z_1^n = i|\vec{O}, A_{sf}^{\theta}, B^{s-1}\right) = \sum_j \tilde{\gamma}_n(i,j) \quad (16)$$

Note that $\alpha_n^{s-1}(z_1^n = i, z_2^n = j)$ and $p(\vec{O}|A_{sf}^{\theta}, B^{s-1})$ were calculated in the Expectation step.

Solving for $\frac{\partial \hat{L}_\lambda}{\partial B_i(k)} = 0$, plug in $\gamma_n(i) \equiv p\left(z_1^n = i|\vec{O}, A_{sf}^{\theta}, B^{s-1}\right)$ and normalizing gives:

$$B_i^s(k) = \frac{\sum_{n=1}^{T}[\delta(o_1^n = k)\,\gamma_n^1(i) + \delta(o_2^n = k)\gamma_n^2(i)]}{\sum_{n=1}^{T}[\gamma_n^1(i) + \gamma_n^2(i)]} \quad (17)$$

Therefore on each step we calculate $\gamma_n(i)$ given α_n^{s-1}, and use equation (17) for the new estimation of the emission matrix (B^s).

These 2 steps repeat until convergence. We then estimate $log\ L(B)$ using the estimated *B*.

Here, during our simulations the agents saw 150 pairs of pictures within each block.

Initiation of B:

Hexagonal grid:

$$B_{z=1}(o = 1) = 1, B_{z=1}(o \neq 1) = 0, B_{z\neq1}(o = 1) = 0,$$

$$B_{z=2}(o = 2) = 1, \qquad B_{z=2}(o \neq 2) = 0, B_{z\neq2}(o = 2) = 0, B_{z=i}(o = j) = p_0\ i,j \neq 1,2$$

$$p_0 = \frac{1}{N_p - 2} \quad (18)$$

Community structure: stayed as above.

The results here are averaged over 30 simulations.

<u>**Supplementary Note 1**: Inference of second level hierarchy</u>

Our community-structure graph had a hierarchical structure, wherein communities are organised on a ring. We hypothesized that inference of the second order structure of a ring and transfer of this structure from day one to day two will allow participants to infer a missing link that closes the ring. We therefore introduced a missing link during the second day. Analysing the results of distance estimation, similarly to experiment one, did not reveal such an effect. Inference of second order structure is harder than a first order structure; we therefore think that one day learning is not enough to reveal this effect.

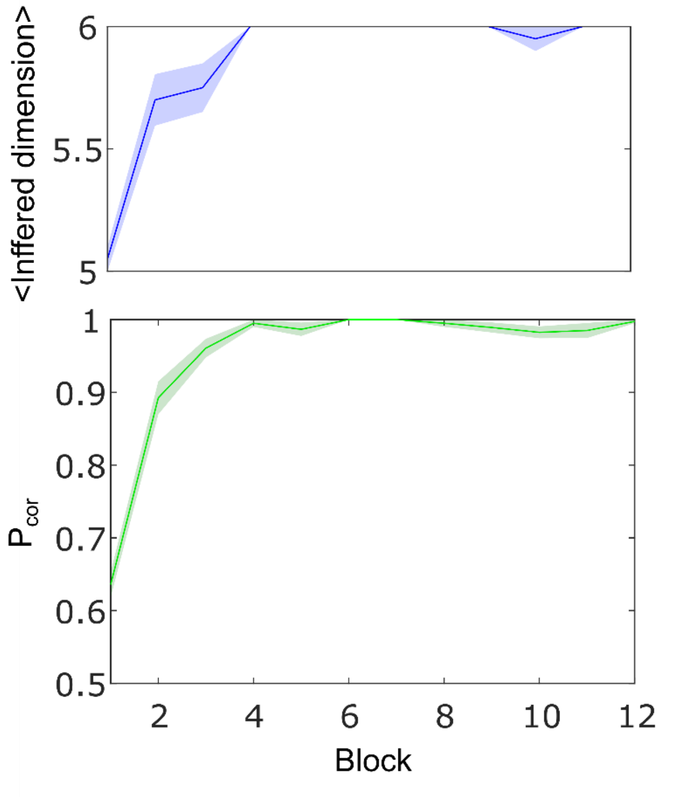

Supplementary Figure1: Learning from random walk - simulations

Learning from random walk on hexagonal graphs, our agent was able to infer the correct structural form and size (upper panel, Y-axis is the inferred number of nodes in one grid dimension, averaged over 15 simulations). The agent determines correctly which of two pictures is closer to a target picture using the approximated transition matrix (lower panel, y-axis is the average fraction of correct responses, out of 60 questions in each block, over 15 simulations).

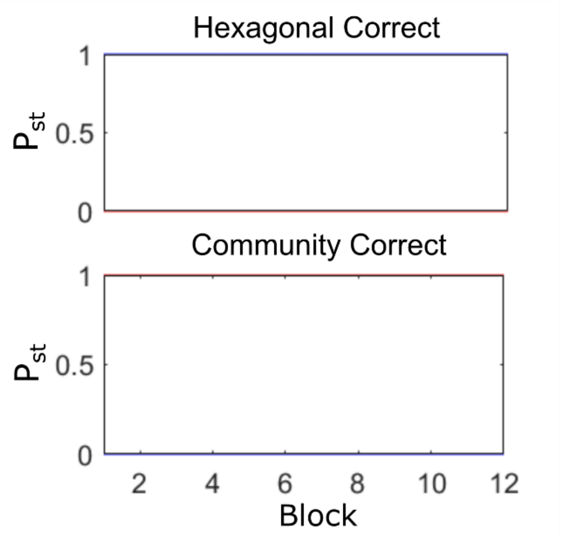

Supplementary Figure 2: Inference of correct structural form was immediate.

Blue: probability of hexagonal structural form. Red: probability for community structural form. Agent learned from random walks.

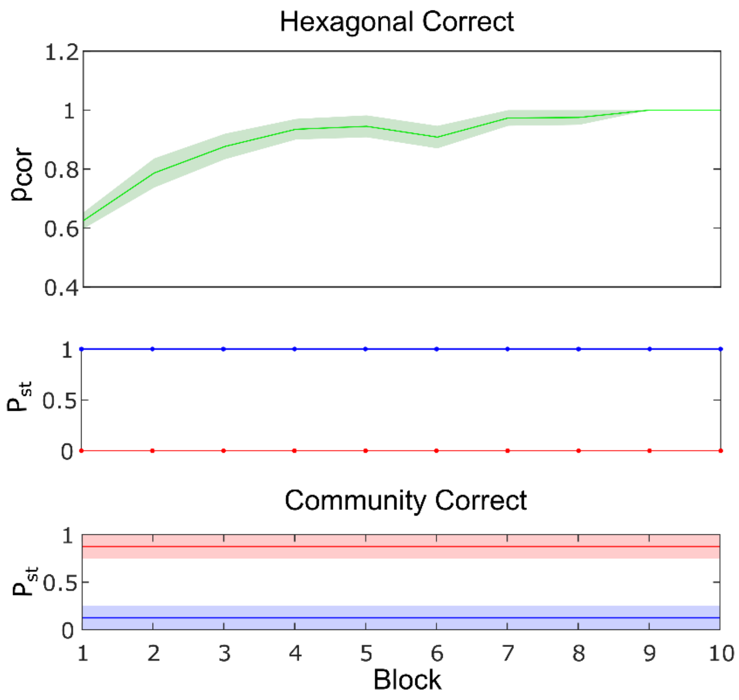

Supplementary Figure 3: Inference on larger and equal size graphs (49x49 graphs)

As the graph sizes were not completely identical, we simulated 49 nodes hexagonal and community structure graphs to show that structural inference was not biased by graph size. Here the agents learned from random walks. The error bands are standard error of the mean. Upper panel: Probability of estimating distances correctly on Hexagonal graphs. Middle panel: inference of structural form (hexagonal correct). Lower panel: inference of structural form (community structure correct).

Supplementary note 2: Border cells:

Entorhinal cortex, the area in which grid cells exist, also has border cells. These cells are active when the animal is near a border [1]. Here we show that changing the behavioural policy of the agent from random walk/ uniform over the graph, into a policy that 'prefers' staying near the borders results in transition matrix eigenvectors that both resemble grid (Supplementary Figure 4B) cells but also border cells (Supplementary Figure 4A). This suggests that border cells, similarly to grid cells, may be part of a basis set which represents tasks on translational invariants graphs (with borders).

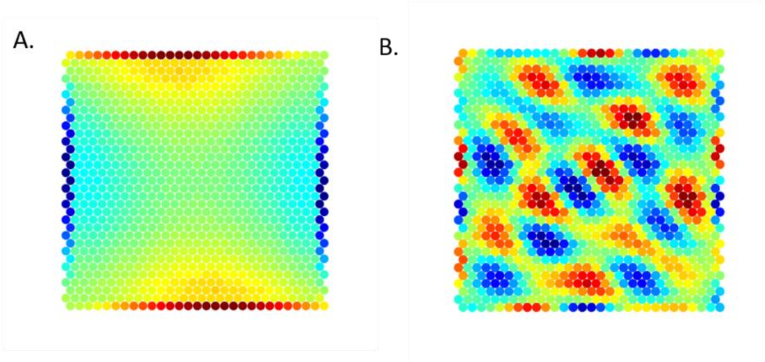

Supplementary Figure 4: Eigenvectors of a transition structure with a Policy of 'prefer borders' have EC border cells pattern.

Here we simulated an agent that wishes to stay near a boundary; the agent probability to move to a node on the boundary was 15 times larger than to move out of the boundary.

A. *An example for an eigenvector with boundary cell pattern*
B. *An example for an eigenvector with hexagonal grid pattern.*

Supplementary Note 3: Inspiration for the choice of basis set for graphs with community structure:

Examples of informative eigenvectors of our community graph transition structure are shown on Supplementary Figure 5A. It can be seen that the eigenvectors are divided into two types; one type encodes the communities and the other encodes connecting node identities. Closure inspection reveals a hierarchical representation: the eigenvectors that encodes the communities are not encoding it simply. Rather, as the communities are organized on a ring, the eigenvectors are a discretized sine wave such that each community is a node on the discretized sine. For better visualization, we have plotted a graph with a larger number of nodes (Supplementary Figure 5B). This observation led us to choose the basis set for community structure as described in the main text. In more details: each node is assigned a number (vector *Vn*), then a connecting nodes vector is determined as following:

$$Vc(n) = exp(-\frac{(Vn - N_n \cdot N_c - 0.5)^2}{2\sigma^2})$$

If the node (*n*) does not belong to the last community. N_c is the community number, $N_n$is the number of nodes in a community, σ the standard deviation of the Gaussian (here $\sigma = 0.5$).

If the node belongs to the last community:

$$Vc^0(n) = exp(-\frac{(Vn_{end} - N_{all} - 0.5)^2}{2\sigma^2})$$

$Vn_{end} = 1\colon N_{all} + N_n$ where N_{all} is the total number of nodes. Then:

$$Vc(1\colon N_n) = Vc^0(N_{all} + 1\colon N_{all} + N_n)$$

$$Vc(N_n + 1{:}N_{all}) = Vc^0(N_n + 1{:}N_{all})$$

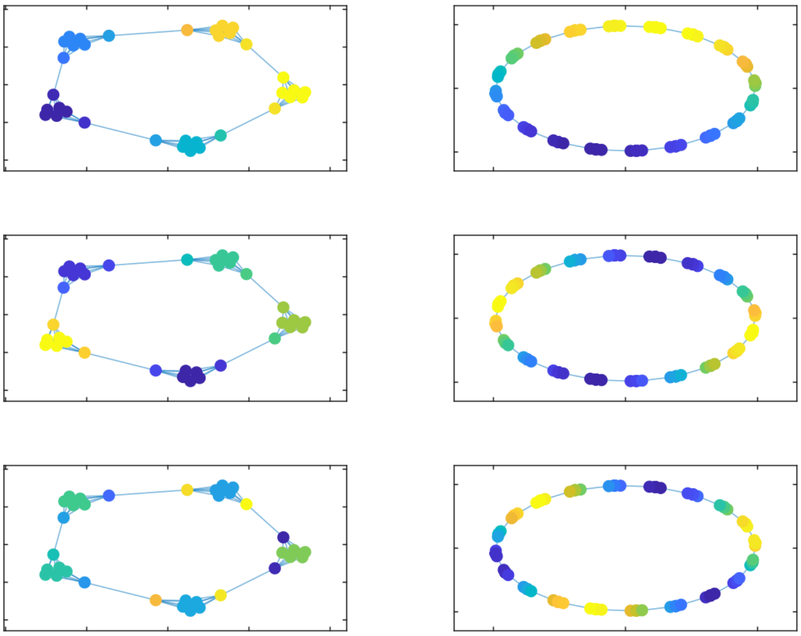

Supplementary Figure 5: Eigenvectors of community structure on a ring.

A) Examples of eigenvectors of the transition structure of a five communities graph. Upper panels assign a community identity while the lower panel assign connective nodes identity

B) Examples of eigenvectors of a transition structure of a larger graph with 20 communities.

Supplementary note 4: Basis set for representing a hierarchical structure**:**

In the main manuscript, we discussed mainly two structural forms; hexagonal grid and community structure. The idea of basis sets can be extended to any other structural form. Here we show that eigenvectors of hierarchical graph transition matrices have interesting patterns that encode the layer information on the hierarchical graph. Similar to abstract representation of connecting nodes and borders, abstract representation of the hierarchical graph layers can support the understanding of the semantic meaning of the layers and the node at the head of the hierarchy.

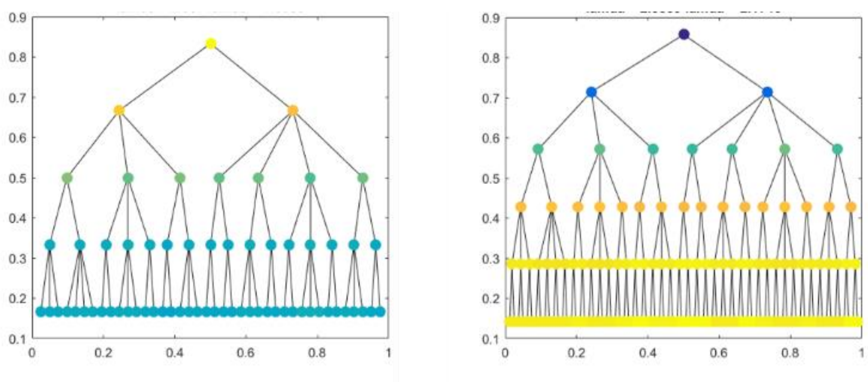

Supplementary Figure 6: Hierarchical graph transition matrix eigenvectors that represent layers information:

Here we show two examples of eigenvectors that represent the distance from the head node.

Supplementary note 5: Human results in learning associations on the graph

Participants' knowledge of the associations were much better than chance (tasks part 2 & part 3 p<<0.001) although the graphs were very large (35, 36 nodes) with a degree of six.

Prior expectation over the structural forms affects learning, such that prior experience of a graph structure confers a slight advantage on even learning basic associations in a new graph of the same structure (Supplementary Figure 7). This in itself is evidence for the influence of prior structural knowledge (here during learning). This effect on learning cannot account for the performance in our distance estimation task (main text Figure 5) as this requires inference of transitions that are never observed. However, if this structural inference *during learning* were combined with an independent regularisation mechanism on the learnt representations (see main text Figure 4B), this may alleviate the requirement for structural inference *during distance estimation*.

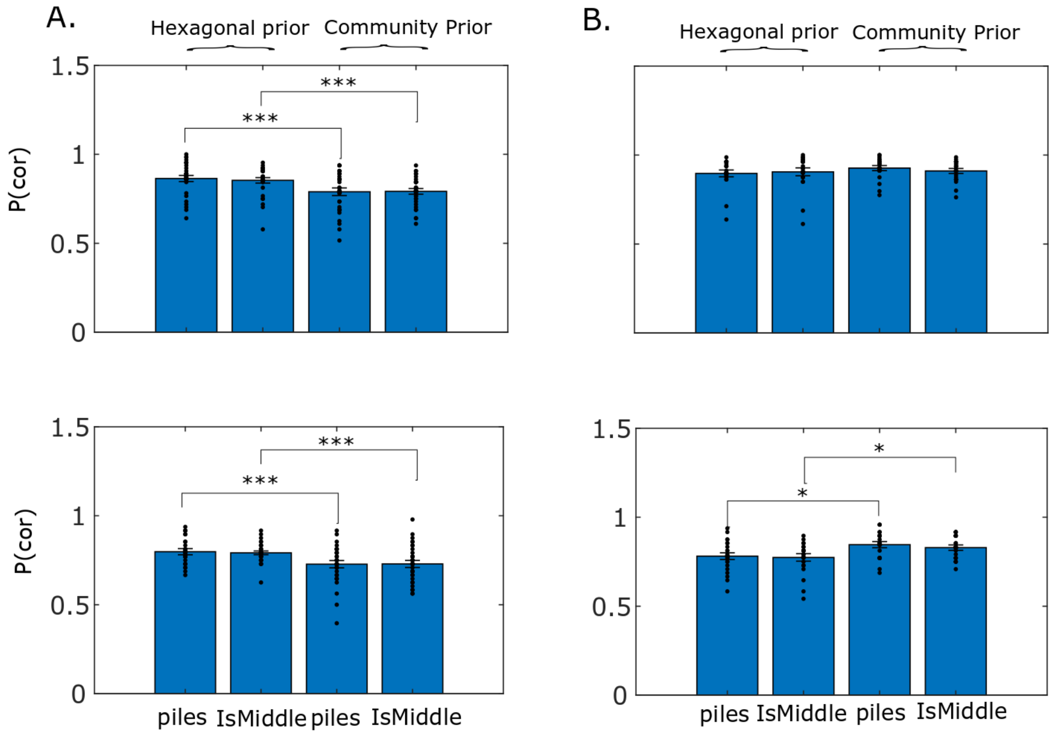

Supplementary Figure 7: Learning pairwise associations: (questions type 2 and 3 in each block), second day

*A) Pairwise associations' knowledge, first experiment. Upper panel trials 4-7, lower panel trials 1-3. Prior expectation over structural form effect learning. Error bars are SEM with average as the center, *** is for p<0.005 (one tailed ttest). Number of participants on each group is 30.*

*B) Pairwise associations' knowledge, second experiment. Upper panel trials 4-7, lower panel trials 1-3. Learning associations is affected by prior expectation over structural form at the beginning of the day but not later. Error bars are SEM with average as the center, * is for p<0.05 (one tailed ttest). Number of participants on each group is 20.*

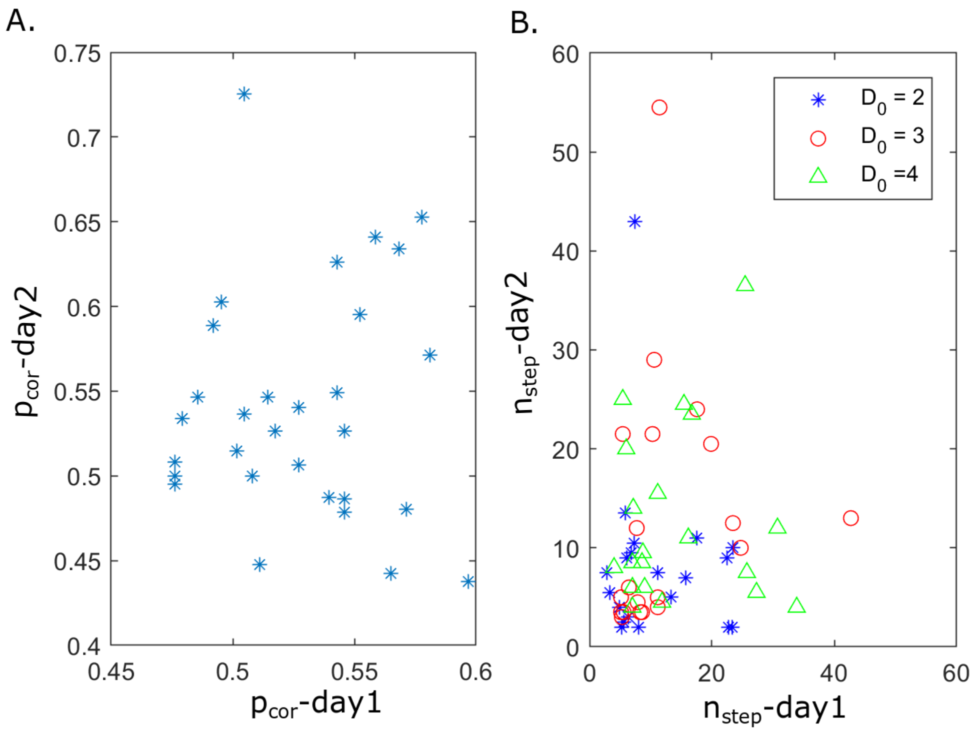

Supplementary Figure 8: Correlations in task performance between day 1 and day 2

A) Fraction of correct distance estimation in day 1 as a function of day 2. There is no significant correlation.

B) Number of steps to the target during day 2 compared to day 1. No significant correlations. (D_0 = 2 C = -0.09, D_0 = 3 C= 0.16, D_0 = 4 C = 0.04).
