# Supplementary material for: Transferring structural knowledge across cognitive maps in humans and models
Source: Nat Commun. 2020 Sep 22;11:4783. doi: 10.1038/s41467-020-18254-6 (PMC7508979; doi:10.1038/s41467-020-18254-6)
Supplement: Supplementary file 4 — Source Data [file 41467_2020_18254_MOESM4_ESM.zip › 233376_2_data_set_4825184_qqwqqz/DataAndCode/DataFileExplanationNew.docx]

Figure 5: Distance estimation

Sheets name: D-estimationXprior, X can be Hex for Hexagonal or Com for community structure.

These arrays contains 0,1 (incorrect/correct) for each questions. 45 questions per block. 35 first questions on each block are inference questions.

Each row is a participant, (0,1) for (incorrect/correct) answer

Figure 6B: RT

Mean log(RT) for each participant.

Each column:

RThexPrior – participants who learned Hexagonal graphs during the first day.

RTcomPrior – participants who learned Community structured graphs during the first day.

Figure 6B: Navigation

There is a table for each prior, for each experiment. Sheet’s name ending with Hex – first experiment, Sheet’s name ending with Com – second experiment. The label Hex/Co before is the prior (first day graph’s structure). Variables names on first row. Each row is a step in the trial that is written on the second column. A trial is finished when participant reach the target. Map 0 – first hexagonal graph, map 1 – second hexagonal graph, map 2 – frist community structure graph, map 3 – second community structure graph. Numbers of choices are nodes on the graph.

Pairwise knowledge data – part 2 &3 on each block – supplementary figure 7

There is a sheet for each experiment and each prior, as before. Each row is a participant and each column is a question. Zero – wrong answer, one – correct answer.
